# Identification of biological mechanisms underlying a multidimensional ASD phenotype using machine learning

**DOI:** 10.1038/s41398-020-0721-1

**Published:** 2020-01-28

**Authors:** Muhammad Asif, Hugo F. M. C. Martiniano, Ana Rita Marques, João Xavier Santos, Joana Vilela, Celia Rasga, Guiomar Oliveira, Francisco M. Couto, Astrid M. Vicente

**Affiliations:** 1 Instituto Nacional de Saúde Doutor Ricardo Jorge, Avenida Padre Cruz, 1649-016 Lisboa, Portugal; 2 Faculdade de Ciências, BioISI - Biosystems & Integrative Sciences Institute, Universidade de Lisboa, Lisboa, Portugal; 3 Faculdade de Ciências, LASIGE, Universidade de Lisboa, Lisboa, Portugal; 4 Unidade de Neurodesenvolvimento e Autismo (UNDA), Serviço do Centro de Desenvolvimento da Criança, Centro de Investigação e Formação Clínica, Hospital Pediátrico, Centro Hospitalar e Universitário de Coimbra, Coimbra, Portugal; 5 Faculty of Medicine, Institute for Biomedical Imaging and Life Sciences, Universidade de Coimbra, Coimbra, Portugal; 6 Faculty of Medicine, University Clinic of Pediatrics, University of Coimbra, Coimbra, Portugal

**Keywords:** Autism spectrum disorders, Personalized medicine

## Abstract

The complex genetic architecture of Autism Spectrum Disorder (ASD) and its heterogeneous phenotype makes molecular diagnosis and patient prognosis challenging tasks. To establish more precise genotype–phenotype correlations in ASD, we developed a novel machine-learning integrative approach, which seeks to delineate associations between patients’ clinical profiles and disrupted biological processes, inferred from their copy number variants (CNVs) that span brain genes. Clustering analysis of the relevant clinical measures from 2446 ASD cases in the Autism Genome Project identified two distinct phenotypic subgroups. Patients in these clusters differed significantly in ADOS-defined severity, adaptive behavior profiles, intellectual ability, and verbal status, the latter contributing the most for cluster stability and cohesion. Functional enrichment analysis of brain genes disrupted by CNVs in these ASD cases identified 15 statistically significant biological processes, including cell adhesion, neural development, cognition, and polyubiquitination, in line with previous ASD findings. A Naive Bayes classifier, generated to predict the ASD phenotypic clusters from disrupted biological processes, achieved predictions with a high precision (0.82) but low recall (0.39), for a subset of patients with higher biological Information Content scores. This study shows that milder and more severe clinical presentations can have distinct underlying biological mechanisms. It further highlights how machine-learning approaches can reduce clinical heterogeneity by using multidimensional clinical measures, and establishes genotype–phenotype correlations in ASD. However, predictions are strongly dependent on patient’s information content. Findings are therefore a first step toward the translation of genetic information into clinically useful applications, and emphasize the need for larger datasets with very complete clinical and biological information.

## Introduction

Autism Spectrum Disorder (ASD) is a neurodevelopmental disorder that manifests with persistent deficits in social communication and interaction, and unusual or repetitive behavior and/or restricted interests^1^. ASD presents a highly heterogeneous clinical phenotype and frequently co-occurs with other comorbidities, such as intellectual disability (ID), epilepsy, and attention-deficit hyperactivity disorder (ADHD)^2–6^. The origin of this clinical variability is unclear, consistent with the absence of reliable diagnostic or prognostic biomarkers. ASD is diagnosed through neurodevelopmental and behavioral assessment, with diagnostic criteria maximizing clinical consensus but of insufficient prognostic value to provide a precise direction for effective intervention for each patient. Improving early diagnosis and prognosis, by using biological markers with a robust predictive power, would provide an advantage to young patients, who benefit the most from an early start of specific intervention^7^. The identification of biomarkers would also contribute to a biological explanation for the clinical heterogeneity of ASD.

Heritability estimates indicate a strong genetic influence on ASD etiology^8–10^. To this day, a large number of genes have been implicated in this disorder, presenting many individually rare variants that overall explain a large proportion of its genetic variance. Structural or sequence genetic alterations can be identified in 20 to 25% of ASD cases^11^, and screening for copy number variants (CNVs) or single-nucleotide variants (SNVs) is nowadays broadly used for etiological diagnosis. The wide genetic heterogeneity that characterizes ASD likely contributes to phenotypic variability in patients^12^, but is still poorly understood. Integrative pathway and network analysis of large-scale ASD genomic studies has advanced significantly the identification of disrupted biological processes in this disorder^13–18^. However, solid models that match these underlying biological processes with clinical phenotypes are still not available, and our understanding of the biological meaning of the large number of putative pathogenic genetic variants, their phenotypic manifestations, and the reliable interpretation of many genetic findings for clinical application is lagging.

Given the clinical and genetic heterogeneity of ASD, it is predictable that a high-impact biomarker will only be relevant for a subgroup of individuals and not the whole patient population. Multiple approaches have been proposed to identify ASD subgroups with similar phenotypic characteristics. Namely, unsupervised machine learning-based stratified models have been employed to stratify patients into subgroups and define ASD subphenotypes. Stratified models use clustering methods, such as hierarchical clustering, to extract data-driven patterns from phenotypic observations that can group individuals with similar characteristics. Since they are independent from prior knowledge and assumptions, unsupervised learning methods have become an important tool for patient stratification, and are used to understand autism heterogeneity. For instance, hierarchical clustering has been applied to a variety of phenotypic measurements, including clinical reports with multiple variables^19,20^, main diagnostic instruments like the Autism Diagnostic Interview-revised, (ADI-R)^21,22^, specific autism-related ability tests like the Reading the Mind in the Eyes Test (RMET)^23^, or neuroimaging data^24^. Overall, these studies defined multiple phenotypic subgroups, which vary with the clinical and behavior parameters analyzed, the study design, the sample sizes, and other factors. Importantly, these studies reinforce the notion that the heterogeneity of autism can be addressed by using machine-learning methods to define meaningful phenotypic subgroups in ASD.

Multidimensional phenotypic subgroups will be fundamentally important for prognosis and treatment of ASD: while it is clear that the current intervention approaches do not benefit equally all patients, it is currently very difficult to predict the course of disease, and what may work better for each individual. To improve this prediction, the patterns that can be identified from a multidimensional phenotypic assessment using machine learning can be associated with disease trajectories and treatment outcomes, highlighting factors mediating treatment response. On the other hand, understanding the biology underlying specific phenotypic clusters will help to identify prognostic biomarkers and define drug targets that work for particular subgroups of patients, and thus improve precision medicine clinical trials.

Mapping phenotypic clusters to biological processes disrupted by genetic variants, to understand the biological meaning of the large number of pathogenic variants identified in ASD and their phenotypic manifestations, has so far been seldom addressed. This would, however, significantly advance the reliable interpretation of many genetic findings for clinical application, improving our understanding of the physiopathology underlying specific phenotypic subgroups of patients. To further our ability to infer clinical meaning from rare CNVs in ASD, for eventual application as biological markers, we developed a machine learning-based approach involving the integration of gene functional annotations and clinical phenotypes. Our approach was developed in four steps, namely (1) definition of phenotypically distinct subgroups in ASD cases; (2) discovery of functionally enriched biological processes defined by rare CNVs disrupting brain-expressed genes in the same ASD cases; (3) assessment of the contribution of disrupted biological processes for classification of ASD phenotypes; (4) design and characterization of predictive effectiveness of a machine-learning classifier for clinical outcome in ASD patients.

## Methods

**Fig. 1:**
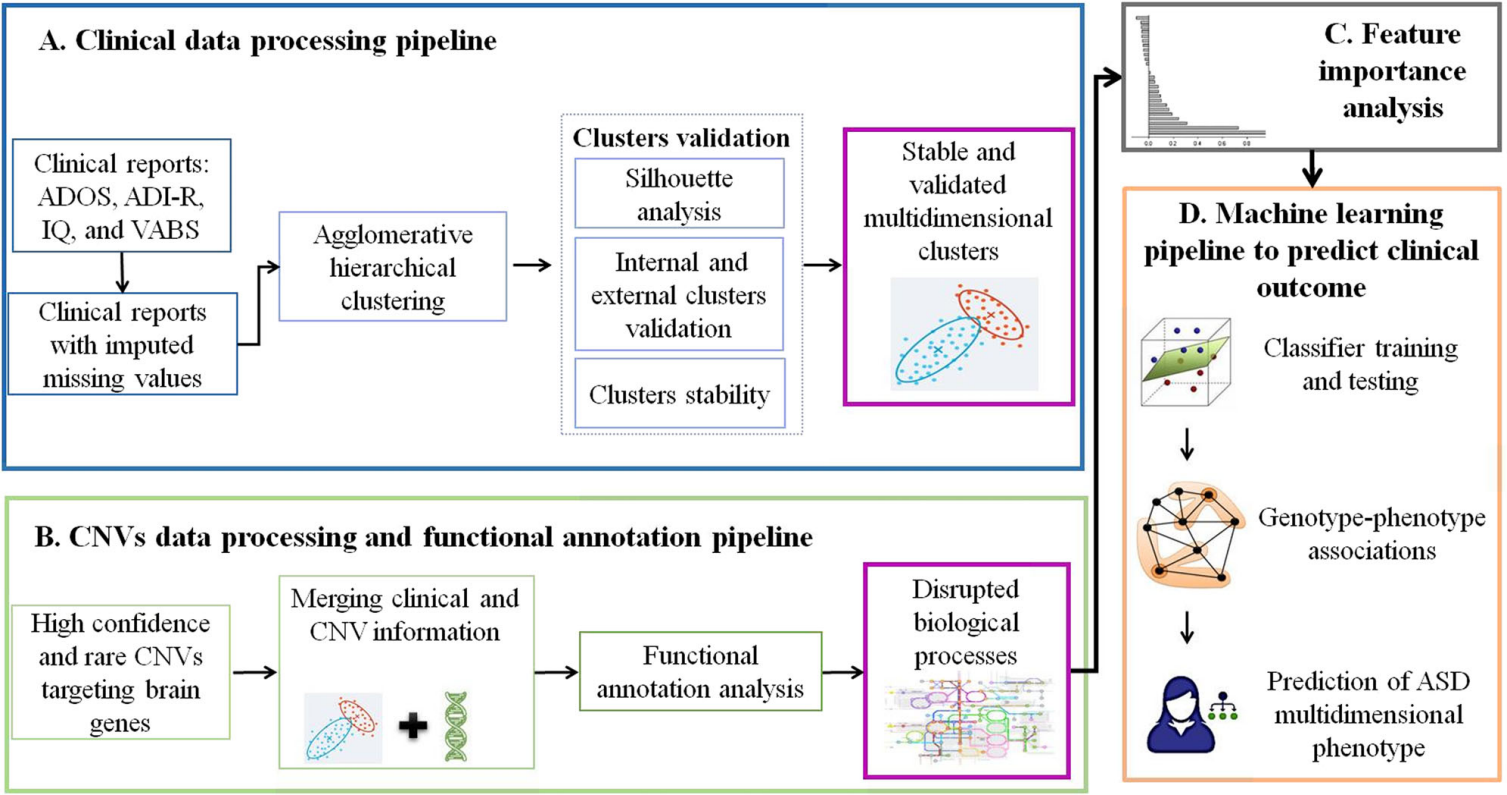
Integrative systems medicine approach to identify complex genotype–phenotype associations. Clinical and genetic data from the Autism Genome Project (AGP) were used in this study. **a** Clinical data analysis processing: clinical data comprise reports of ASD diagnosis and neurodevelopmental assessment instruments. Agglomerative hierarchical clustering (AHC) was used to identify clinically similar subgroups of individuals in stable, validated clusters, defined by multiple clinical measures. **b** CNV data processing: rare high-confidence CNVs previously identified by the AGP, targeting brain-expressed genes, were retained for analysis. CNV data were merged with clinical data from clustered ASD subjects for a final list of CNVs targeting brain genes. **c** Functional annotation analysis: biological processes defined by brain-expressed genes targeted by CNVs were obtained by using g:Profiler. **d** Classifier design: a Naive Bayes machine-learning classifier was trained and tested on patient’s data, to predict the phenotypic clustering of patients from biological processes disrupted by rare CNVs targeting brain-expressed genes.

Figure 1 shows the graphical representation of the overall methodology, described in detail below.

### Participants

The ASD dataset used in this study was obtained from the Autism Genome Project (AGP)^25^ database, and comprises CNV data and clinical information from 2446 ASD patients. The AGP was an international collaborative effort from over 50 different institutions to identify risk genes for ASD. The group of individuals with phenotypic information from clustering and rare CNV data, used in final analysis, included 1213 males (83.4%) and 144 females (10.6%). Approval was obtained from the ethical committees of all the institutions participating in the AGP consortium, and informed consent was granted by all subjects or their parents/legal guardians.

### ASD diagnosis, clinical assessment instruments, and clinical features

Individuals meeting criteria defined by the Diagnostic and Statistical Manual of Mental Disorders IV (DSM-IV)^26^ and the thresholds for autism or ASD from the Autism Diagnostic Interview-Revised (ADI-R)^27^ and the Autism Diagnostic Observation Schedule (ADOS), were classified as ASD cases^28^. The AGP defined a phenotypic classification system based on the combined ADI-R and ADOS diagnosis, categorizing subjects into Strict (meeting thresholds for autism by the ADI-R and ADOS), Broad (meeting thresholds for autism from one instrument and ASD from the other), and Spectrum (meeting thresholds for autism from at least one instrument or ASD from both). Individuals with an ASD diagnosis from only one instrument and no information from the other, or not meeting thresholds for autism or ASD from one of the instruments, regardless from the classification from the other, were not included in the study. Clinical measures used in this study were retrieved from the AGP database, including the ADI-R verbal status, ADOS severity score, Vineland Adaptive Behaviour Scales (VABS)^29^ subscales, and an Intelligence Quotient (IQ).

The ADI-R verbal status is a dichotomized measure indicating the verbal status of the individual at evaluation. The ADOS severity metric ranges from 1 to 10, and is calculated from ADOS modules 1–3 raw scores^30^. As there is no algorithm available to calculate ADOS severity score for ADOS module 4 reports, which is applied only to adolescents and adults, subjects with the ADOS module 4 (*N* = 149) were dropped from further processing. The severity score distribution is collapsed into three categories, namely Autism (severity scores ranging from 6 to 10), ASD (severity scores ranging from 4 to 5), and Non-Spectrum (severity scores from 1 to 3), which reflect the mapping of the severity metric onto raw ADOS scores. The ADOS Non-spectrum category includes individuals with a mild phenotype, and in this study 125 individuals with a Non-spectrum ADOS severity score fell within the Spectrum phenotypic class from the AGP, meaning that they met thresholds for autism from the ADI-R, and were thus included.

The VABS is used to assess adaptive functioning of individuals, and consists of three subscales, namely, socialization, communication, and daily living skills scores, and also computes a composite score. Subjects with VABS scores ≤ 70 were classified in a dysfunctional adaptive behavior category, for all subscales. IQ scores of ASD cases were also retrieved from the AGP database, and categorized with the following thresholds: IQ > 70 normal, 50 < IQ < 70 mild intellectual disability, and IQ < 50 severe intellectual disability.

Clinical reports from the ASD patients were examined for missing values, and clinical features with more than 70% information were retained for the analysis. To minimize missing value imputation bias, individuals with missing values above this threshold for more than two clinical features were also excluded. Completeness of each clinical feature is reported in Table S1 (Supplementary File 1). Missing values were imputed using the missForest^31^ R package that implements the Random Forest^32^ algorithm, a decision tree-based supervised machine-learning method. Imputation error was assessed using the normalized global Proportion of Falsely Classification (PFC), and the missing value imputation error was 0.12.

### Clustering analysis of ASD clinical data

To focus on core domains of ASD symptoms, verbal skills, disease severity, adaptive behavior, and intellectual levels, which strongly condition prognosis, were selected for further analysis. Verbal status was obtained from the ADI-R, ASD severity scored from the ADOS, adaptive functioning from the VABS, using its three subdomains, and a performance IQ category from the IQ assessment contributed by participating sites to the AGP database. Other IQ domains had too many missing values to be used. The Agglomerative Hierarchical Clustering (AHC)^33^ method was used to identify independent phenotypic subgroups from the selected clinical features. Correlations between clinical features were assessed using the Pearson method, and features with a correlation value of >0.75 were considered correlated. The Gower^34^ metric was used to calculate the distance matrix from the patient’s clinical data. To normalize the effect of highly correlated variables on clustering, the weight for correlated variables (VABS subscales of socialisation, communication, and daily living skills) was reduced to half during distance matrix calculation. To identify phenotypic subgroups, the AHC method using Ward2^35^ criteria was applied to the distance matrix.

To assess the contributions of each clinical feature in defining the clusters, we excluded one feature at a time, re-performed the clustering, and observed the changes in Silhouette values of both clusters. For this purpose, we selected Silhouette value as an evaluation metric because it was also used to define outliers in clinical data. A decrease in the Silhouette value of a cluster after removing one feature indicates its importance in defining this cluster and vice versa.

### Goodness of clustering assessment

A Silhouette method^36^ was employed to estimate the goodness of the clustering results. The Silhouette value for each individual shows how well the individual is clustered, and ranges from −1 to 1, with individuals scoring below 0 considered as wrongly clustered. In addition, the Silhouette value for each cluster was derived, and clusters with Silhouette value of >0.25 were considered as true clusters. Bootstrapping with 1000 iterations was used to measure the stability of clusters, where a boot mean value above 0.85 corresponds to stable clusters. All clustering analysis was performed in R environment, using Cluster^37^ and FPC packages.

### Functional enrichment analysis

Genotyping and CNV calling methods for the AGP ASD subjects (*N* = 2446) were previously described^25^. CNVs called by any two algorithms (high-confidence CNVs) and above 30 kb in size were retained for further analysis. To screen for rare CNVs (<1% in control population), CNV frequencies in control populations were estimated using the genotypes from the studies by Sheikh et al.^38^ (*N* = 1320) and Cooper et al.^39^ (*N* = 8329), identified using the same genotyping platform^25^. Control genotypes were obtained from the Database of Genomic Variants (DGV)^40^.

To focus CNV selection on variants spanning brain-expressed genes, avoiding a priori hypotheses from ASD candidate gene assumptions, an extensive list comprising 15585 brain-expressed genes was obtained from Parikshak et al.^41^. The brain-expressed gene list was prepared from brain RNA-seq data, collected at 13 different developmental stages, including genes expressed during the early brain developmental phase. The full criteria and parameters used to define the brain-expressed gene list were previously described^41^.

The g:Profiler^42^ tool was employed to identify biological processes enriched for brain-expressed genes spanned by rare CNVs in ASD individuals. g:Profiler implements a hypergeometric test to estimate the statistical significance of enriched biological processes, followed by multiple corrections for the tested hypotheses using the Benjamini–Hochberg procedure. g:Profiler uses Gene Ontology (GO) data to find the biological annotations for input genes.

The GO tool contains a Directed Acyclic Graph (DAG) structure with a clear hierarchical parent-to-child relationship between GO terms. Because of this DAG structure, functional enrichment analysis can result in redundant GO terms, which may lead to high correlations between GO terms. To minimize the correlations between GO terms, the Revigo^43^ tool was employed to redundant GO results. Revigo uses the methods of semantic similarity to measure similarities between GO terms. The SimRel^44^ method was used to calculate similarities between GO terms, and terms with a similarity score of >0.7 were grouped.

### Feature importance assessment

The mean decrease in the accuracy of the Random Forest algorithm was used to compute the importance score of each disrupted biological process for categorizing ASD subjects into defined phenotypic clusters. A stratified tenfold cross-validation quantifies the importance of all features. The importance score of all disrupted biological processes was recorded at each fold. A final importance score for each biological process was calculated by averaging their importance score values across all the ten folds. Random Forest was implemented using randomdForst R package^45^.

### Classifier training and cross-validation

A machine-learning binary classification method was employed to predict ASD phenotypic groups, previously defined by the clustering analysis, from biological processes disrupted by rare CNVs. The specific method used was the Naive Bayes classifier^46^ (Table S4, Supplementary File 1). The method was implemented in the klaR R package, with default parameters. Precision, recall, specificity, and F score were used as evaluation measures. To train and test the classifier, a stratified fivefold cross-validation approach was applied, in which data were first split into five equal subsets with equal class probabilities; the classifier was trained on any four subsets, and the remaining subset was used as the test set. This process was repeated five times, and each time a different subset was used as the test set. For each repetition, the model performance was estimated, and the mean values for precision, recall, specificity, and F score were reported. The classifier was trained on patient’s data by using the “more severe” cluster as the positive class and the “less severe” cluster as the negative class.

The information content (IC) from each individual represents the level of specificity of biological process disruption, and was derived by summing the IC values of all the biological processes disrupted in each individual. IC is a numerical value that describes the specificity of a GO term by using its position in the GO DAG structure.

## Results

### Identification of ASD clusters defined by clinical phenotype

A total of 1817 ASD subjects from the AGP were retained for analysis after assessment of missing values in clinical features. Agglomerative hierarchical clustering analysis of clinical observations from these patients initially identified two phenotypically independent clusters. To minimize the phenotypic complexity and define the most stable and cohesive clusters, weakly clustered individuals with a Silhouette value <0.300 (representing a balance between the number of individuals lost and goodness of clustering) were excluded from the clustering analysis. After removal of weakly or wrongly clustered individuals, cluster 1 contained 903 ASD cases, while cluster 2 comprised 494 patients (Table 1). Elimination of the loosely clustered individuals resulted in more stable and cohesive clusters, with high values for cluster stability, and reduced the average distance between the two individuals in a cluster (Table 1). Overall, the cluster validation through the Silhouette method and bootstrapping showed that both clusters were true and consistent.

**Table 1:** Clustering validation, after removal of weakly clustered individuals.

Cluster validation measures	Cluster 1	Cluster 2
Cluster size (*N*)	903	494
Average distance between two patients	0.235	0.231
Silhouette value	0.567	0.579
Average Silhouette of both clusters	0.571	
Cluster stability	0.998	0.996

### Clinical interpretations of the clusters

All clinical measures differed significantly between the two clusters, as shown in Table 2. Cluster 1 (Supplementary File 1: black circles in Fig. S1) includes a higher number of individuals, who generally exhibited a milder clinical phenotype, while Cluster 2 (Supplementary File 1: red triangles in Fig. S1) included a higher percentage of subjects with severe dysfunction. All individuals in Cluster 1 were verbal according to the ADI-R, while Cluster 2 included only nonverbal cases. The mean age of ADI-R assessment was 7.7 years, an age when verbal status is generally well established. Furthermore, the mean age of individuals in Cluster 1 (mean age 8.02) and Cluster 2 (mean age 7.01) did not significantly differ.

**Table 2:** Clusters 1 and 2 statistics for each clinical measure.

Clinical measure	Clinically defined categories	Cluster 1 *N* (%)	Cluster 2 *N* (%)	*p* value
ADI-R verbal status	ADI-R-nonverbal	0 (0)	494 (100)	<0.00001^a^
	ADI-R-verbal	903 (100)	0 (0)	
ADOS severity score	ADOS severity score Autism (score 6–10)	714 (79.07)	392 (79.35)	<0.00001^b^
	ADOS severity score ASD (score 4–5)	64 (7.09)	102 (20.65)	
	ADOS severity score Non-spectrum (score 1–3)	125 (13.84)	0 (0)	
VABS communication	Dysfunctional VABS communication (score ≤ 70)	307 (34)	493 (99.8)	<0.00001^a^
	Normal VABS communication (score > 70)	596 (66)	1 (0.2)	
VABS daily living skills	Dysfunctional VABS daily living skills (score ≤ 70)	478 (52.94)	484 (97.98)	<0.00001^b^
	Normal VABS daily living skills (score > 70)	425 (47.07)	10 (2.02)	
VABS socialization	Dysfunctional VABS socialization (score ≤ 70)	497 (55.04)	490 (99.19)	<0.00001^a^
	Normal VABS socialization (score > 70)	406 (44.96)	4 (0.81)	
Performance IQ Scale	Severe disability (score < 50)	2 (0.22)	218 (44.13)	<0.00001^b^
	Moderate disability (score ≥ 50 and ≤ 70)	31 (3.43)	125 (25.3)	
	Normal ability (score > 70)	870 (96.35)	151 (30.57)	
Gender	Male	830 (91.92)	417 (84.41)	0.000015^b^
	Female	73 (8.08)	77 (15.59)	

^a^Fisher’s exact test.

^b^Chi-square test.

For all VABS subdomains, roughly half of the subjects in Cluster 1 were in the normal range; conversely, over 97% of individuals belonging to Cluster 2 showed dysfunctional adaptive behavior. Consistent with the other clinical measures, over 96% of cases from Cluster 1, but less than one-third in Cluster 2, scored at the normal level in performance IQ, while a much higher percentage of ASD cases from Cluster 2 than from Cluster 1 presented with a performance IQ in the range of severe intellectual disability.

Regarding the ADOS severity score, ~14% of the individuals in Cluster 1 were assigned to the milder category of the ADOS severity score (“Non-spectrum” for ADOS, but scoring positive for “Autism” in the ADI-R, and therefore classified in the AGP “Spectrum” phenotypic class, see “Methods”). Conversely, none of the individuals in Cluster 2 scored in this category. On the other hand, a significantly higher percentage of cases in Cluster 2 (20.65%) than individuals in Cluster 1 (7.09%) scored in the intermediate ASD severity category. It is noteworthy that both clusters show a similarly high percentage of individuals scoring in the “Autism” ADOS severity category. This is not surprising since this broad category (scores ranging from 6 to 10) comprises all subjects classified in the Strict AGP phenotype class, but also a large proportion of individuals in the AGP Broad phenotype class. The “Autism” ADOS severity score therefore targets a subset of the study population that can be quite heterogeneous in phenotypic presentation. Corroborating this, we found that the “Autism” category of the ADOS severity score is not significantly associated with the clusters (*χ*^2^ = 0.15, *p* = 0.901, df = 2), even though overall there is a significant association of the overall ADOS severity scores (Table 2). Both clusters were strongly dominated by the male gender, partly because of the high percentage of males in the dataset after the elimination of weakly or wrongly clustered individuals. However, the percentage of males was higher in cluster 1, representing the milder phenotype, consistent with general observations that male-to-female ratios are higher in datasets that comprise more high-function ASD individuals.

Analysis of the contribution of each clinical feature in defining clusters showed that the main contributor was the ADI-R verbal status variable (Supplementary File 1: Table S2). The VABS subscales had a strong effect on Cluster 1, but a modest role in defining Cluster 2. Performance IQ also contributed more to Cluster 1, whereas for Cluster 2 it had the least effect. The ADOS severity score did not have a major role in defining either cluster, as indicated by the similar high percentage of subjects scoring within the range of “Autism” in the ADOS severity scale in both clusters. Similarly, gender was not an important contributor to the definition of either cluster.

### Disrupted biological processes from brain-expressed genes targeted by rare CNVs

CNVs (*N* = 129,754) identified in 2446 subjects with ASD were filtered to select rare, high-confidence CNVs, over 30 kb in size and that contained complete or partial brain-expressed gene sequences. The selected high-confidence, rare CNVs (*N* = 12,683) disrupted 4025 brain-expressed genes in 2414 subjects with ASD (86.8% males and 13.2% females).

Phenotypic cluster and rare CNV data were complete for 1357 individuals with ASD, and available for integration. Functional enrichment analysis of rare CNVs targeting brain-expressed genes (*N* = 2738) in 1357 patients identified 17 statistically significant biological processes (Supplementary File 1: Table S3). g:Profiler did not recognize 187 genes from the input list.

The redundancy of GO terms in functional enrichment analysis, caused by overlapping annotations in ancestor and descendent terms in the DAG structure of GO, was reduced by grouping the terms that had a semantic similarity score greater than 0.7 (Supplementary File 1: Table S3). The Revigo tool used to reduce redundancy did not recognize one biological process (Plasma membrane-bounded cell projection organization). After redundancy reduction, 16 biological processes remained (Table 3), with the Calcium-dependent cell–cell adhesion via plasma membrane cell adhesion molecules biological process merged with Homophilic cell adhesion via plasma membrane adhesion molecules (similarity score = 0.76). The most significant biological process identified in this dataset was Homophilic cell adhesion via plasma membrane adhesion molecules, which includes 53 brain-expressed genes disrupted by the selected CNVs. The ten most significant biological processes were related to cell adhesion and cellular organization, and also included nervous system development and protein polyubiquitination (Table 3). Moreover, two significant biological processes were related to behavior and cognition.

**Table 3:** Statistically significant enriched biological processes for CNVs spanning brain-expressed genes (*N* = 2738).

Biological processes	Enriched genes (*N*)	FDR *p* value
Homophilic cell adhesion via plasma-membrane adhesion molecules	53	6.30E–09
Cell–cell adhesion via plasma-membrane adhesion molecules	66	1.70E–07
Cellular component organization or biogenesis	944	5.70E–05
Cellular component organization	915	7.00E–05
Cellular component biogenesis	475	0.00066
Cellular component assembly	434	0.00177
Nervous system development	363	0.00215
Organelle organization	562	0.00475
Protein polyubiquitination	64	0.00592
Cell projection organization	231	0.00836
Cellular localization	418	0.0091
Single-organism behavior	83	0.0196
Regulation of cellular component organization	364	0.0257
Plasma-membrane-bounded cell projection organization	223	0.0282
Cognition	56	0.0364
Single-organism organelle organization	263	0.044

*FDR* false discovery rate.

### Biological process importance for prediction of ASD clinical phenotype

The enriched biological processes and phenotypic cluster information for ASD cases were combined in a matrix to assess the predictive value of the biological processes for categorization in one of the two phenotypic clusters, broadly characterized by a milder and a more severe phenotypic presentation. The 57 individuals, containing both rare CNV and cluster information that did not present any enriched biological process, were excluded, so further analysis comprised 1300 ASD patients.

Table 4 shows the ranking in importance of disrupted biological processes for categorization of subjects into ASD phenotypic clusters, computed using the Random Forest importance score function. The importance of each biological process was calculated using the mean decrease in accuracy, computed by permuting each biological process. The feature importance analysis using Random Forest, which was trained and tested using a stratified tenfold cross-validation over the integrated dataset, revealed positive values for all features, indicating that all of the biological processes are positively contributing for classification. The most important biological process for the classification was Regulation of cellular component organization, with a mean decrease in accuracy of 0.052. The most significantly enriched biological process in the overall ASD dataset, Homophilic cell adhesion via plasma membrane adhesion molecules, was ranked at position 14, indicating that it is not a top contributor to phenotypic categorization of ASD subjects into the phenotypic clusters, in this population.

**Table 4:** Importance of each biological process from random forest in classifying ASD subjects into defined phenotypic clusters.

Random Forest rank	Biological process	Mean decrease in accuracy
1	Regulation of cellular component organization	0.052
2	Cell projection organization	0.025
3	Cellular component assembly	0.025
4	Single-organism behavior	0.020
5	Organelle organization	0.018
6	Single-organism organelle organization	0.017
7	Cellular component biogenesis	0.014
8	Cognition	0.013
9	Nervous system development	0.010
10	Cellular localization	0.009
11	Cellular component organization	0.006
12	Protein polyubiquitination	0.005
13	Homophilic cell adhesion via plasma-membrane adhesion molecules	0.005
14	Cell adhesion via plasma-membrane adhesion molecules	0.005
15	Cellular component organization or biogenesis	0.003

### Predicting clinical phenotype from the biological processes disrupted by rare CNVs in ASD patients

The machine-learning classifier was trained and tested using phenotypic clustering information and the 15 biological processes inferred from rare CNVs targeting brain-expressed genes in ASD patients. The classifier was trained with the assumption that ASD subjects with a more dysfunctional clinical phenotype, subgrouped in Cluster 2, would present a different pattern of disrupted biological processes from the individuals, with a milder expression of ASD phenotype in Cluster 1.

The classifier trained on data from 1300 patients did not perform well in predicting the more dysfunctional clinical phenotype from disrupted biological processes (Table 5), with scores indicating a low accuracy of the predictive model.

**Table 5:** Naive Bayes performance in predicting the severe phenotype of ASD.

Data used for classification	N	Precision	Recall	Specificity	F score
All ASD cases	1300	0.221	0.379	0.655	0.279
ASD cases from the first quantile with the highest IC	**325**	**0.816**	**0.389**	**0.699**	**0.526**
ASD cases from the first and second quantiles of IC	649	0.23	0.384	0.65	0.284
ASD cases from the first three quantiles of IC	974	0.29	0.389	0.672	0.329

To further dissect the information available, the biological process IC for each individual was calculated, by summing the IC values for all the biological processes disrupted in that individual. ASD subjects in the first IC quantile (*N* = 325) had the highest IC scores, while ASD cases belonging to the fourth quantile (*N* = 326) contained the lowest IC scores. The performance of the Naive Bayes classifier improved when only ASD subjects with higher IC were selected for analysis. Analysis of the group of individuals with the highest IC (first quantile) resulted in a higher predictability of ASD clinical outcome (Table 5). The classifier trained and tested on individuals from the first two (first and second) and first three (first, second, and third) quantiles also performed better than the classifier designed by using the whole dataset of clusters and biological processes (Table 5). The Naive Bayes classifier was thus able to make reasonably good predictions of ASD severity, but only for a subset of ASD individuals with higher IC. This indicates that improved GO information, as well as larger datasets with more GO information available, are needed to usefully integrate clinical and biological data.

## Discussion

In spite of the enormous volume of genetic information generated by genomic approaches in the past decade, the diagnosis of ASD patients is still solely based on neurodevelopmental assessment. The results of many genomic tests, including CNV arrays and clinical exomes, still leave about 80% of the cases without any explanation regarding the biological pathways underlying their disease and their personal clinical presentation, with strong implications for adequate therapy.

In this study, we developed a novel integrative approach to predict ASD phenotypes from biological processes defined by genetic alterations. Overall, our approach sought to exploit multidimensional clinical measures to define subgroups of ASD patients presenting similar clinical profiles, and then to identify the biological processes disrupted by CNVs that might predict these more homogeneous clinical patterns. For the sake of eventual clinical utility, we chose clinical measures with well-established relevance and frequently used in clinical settings, but established no other restrictions. Further, we did not set any a priori hypothesis for gene selection, besides being expressed in the brain.

The clustering of clinical data from ASD cases resulted in two subgroups that were clearly distinguishable in terms of severity of phenotype, defined by multiple clinically relevant measures including verbal status, ASD severity, adaptive function, and cognitive ability. The identification of only two clusters for the clinical phenotype, with an important proportion of individuals in the AGP dataset that could not be adequately clustered, was expected, as it reflects the high clinical heterogeneity of ASD. The identification of these two subgroups was in line with previous results by Veatch et al.^19^, who also identified two clusters differing in severity using two independent population samples, including the Autism Genetic Resource Exchange (AGRE) and also the AGP dataset, and several phenotypic measures in common with our study. While clinical variables were not fully coincidental between the two studies, we confirmed that the verbal status, ADOS-based severity, VABS-based communication, socialization, and daily living skills, as well as gender, were all significantly different between clusters. We further noted an unequal contribution of each clinical measure to the definition of the clusters, with verbal status the main contributor and the ADOS severity score a low contributor for both clusters, while Performance IQ was mainly important for Cluster 1.

The larger Cluster 1 was characterized by a generally milder phenotype, with all individuals being verbal, a large proportion in the normal IQ range, and significantly higher numbers of subjects scoring better in adaptive behavior subscales. Cluster 1 also showed a higher male-to-female ratio, as expected given the general observation that higher-functioning ASD subgroups have a larger proportion of males. The smaller Cluster 2 included only nonverbal subjects, and had a higher percentage of subjects with a more dysfunctional phenotype in terms of adaptive behavior, as well as lower IQ scores. Because cognitive ability is such an important variable for prognosis, we included performance IQ as a clinical variable, in spite of the limitations related to the heterogeneity of IQ measurement tools used for patient assessment by AGP-contributing sites. For the AGP dataset, an effort was previously made to rationalize the multiple tests used, and cognitive level was established using a categorical classification provided by AGP sites in three categories, namely severe intellectual disability, mild intellectual disability, and normal IQ, for verbal, performance, and full-scale IQ scores. Limitations were also introduced by the high proportion of missing data; given the adopted control of the validity of imputation procedures, only performance IQ met the criteria for reliable imputation, so only this measure was used.

Because our main goal was to improve the power for phenotypic subgroup prediction by genetically defined biological processes, we focused on obtaining compact and stable clusters by using strict criteria for cluster stability to assess the goodness of clustering, at the expense of population sample dimension. As expected, the weakly clustered individuals tended to have more divergent scores across clinical measures (data not shown), and therefore were more difficult to cluster with high confidence. It is intriguing that a higher proportion of females than males was removed, suggesting that this divergence of scores is more frequent in girls. This observation generally supports recent debates on the lower adequateness of assessment criteria to the female autism phenotype^47^.

Previous clustering studies have been able to identify multiple phenotypic clusters in populations of individuals with autism^19–22^, using different datasets and various study designs with specific goals. Besides the above-mentioned study by Veatch et al.^19^, conducted in the AGRE and AGP populations, Hu and Steinberg^22^ also analyzed nearly 2000 individuals from the AGRE dataset, selecting 123 ADI-R items for clustering analysis, and defining four different subgroups for association analysis of gene expression profiles. Other studies explored different phenotypes, like developmental trajectories at an early stage^19^, or used different study designs to analyze severity gradients within subgroups^20^. The variability in the number of clusters reported in these previous studies is likely explained by the phenotypes used, for instance the analysis of one primary instrument as opposed to multidimensional phenotypic measures; by the study design, e.g., the inclusion criteria for the study set, which might be broader, or else focus on subjects fulfilling strict criteria or high IQ, and by sample size. Given the heterogeneity of the ASD phenotype, the sample size of a study is likely crucial, as in small samples overenrichment of a specific group or strata may be more likely, and inflated effect sizes will restrict replication in other datasets^48^. Lombardo et al.^49^ previously described a simulation analysis to illustrate the issues related to small sample size effect and effect size inflation, which indicated that sample sizes above 1000 subjects could provide a correct estimation of true effect sizes. In this study, clustering analysis of a larger sample (*N* = 1397) yielded two phenotypically distinct subgroups, reinforcing the previous definition of two clusters by Veatch et al.^19^ in two large datasets (AGRE and the AGP), and using overlapping phenotypic measures, and the need of larger sample sizes for meaningful outcomes.

Other types of phenotypic measures have also been used for parsing heterogeneous datasets into clinically and/or biologically meaningful subgroups, and contribute to better designed and more personalized interventions. For instance, Lombardo et al.^23^ analyzed two independent datasets with and without ASD for the ability to read emotions and mental states in the eye region of the face using the RMET. The authors identified five and four distinct profile subgroups in patients and typically developing (TD) controls, respectively, and showed that within ASD, three subgroups were clearly impaired, while two showed results comparable to TD controls, supporting the notion of a continuum of impairments with typical behavior. Another interesting study carried out agglomerative hierarchical clustering to partition neuroanatomical data from ASD subjects into three subgroups, relating to functional networks involved in processes compromised in ASD, like social cognition and interactions and communication^24^. Structural mapping to subgroups was associated with clinical variables (IQ, ADOS severity), showing differential ADOS severity with predictive value. Finally, another data-driven approach, the Similarity Network Fusion (SNF) method, was employed to integrate neuroimaging, social cognitive, neurocognitive, and demographic data across several neuropsychiatric disorders and unaffected subjects, identifying four subgroups with distinct neurocognitive and social circuit profiles^50^. These studies go beyond accepted nosology definitions to identify altered neurobiological circuits that can be targeted for specific treatment according to brain dysfunction.

To test the hypothesis that phenotypic subgroups have specific underlying pathological mechanisms, we sought to identify the biological processes enriched in the gene sets disrupted by rare CNVs detected in the AGP dataset. The functional enrichment analysis conducted in this study was independent of any prior assumptions or weighting criteria of genes relative to ASD risk. To make functional enrichment analysis hypothesis-free and to let the data speak, we screened for CNVs disrupting any brain-expressed genes. The objective was to obtain a complete picture of the convergence of rare CNVs, targeting any brain-expressed genes, into biological processes relevant for brain function.

The biological processes identified in the functional enrichment analysis showed an overlap with putative core biological mechanisms of ASD defined by previous studies. For example, 363 brain genes spanned by rare CNVs were enriched in neurodevelopment biological process, and 56 genes were associated with cognition process. Enrichment of nervous system development and cognition processes in ASD has been previously reported by studies using different approaches, including transcriptome analysis and co-expression networks^15^, and is supported by the function of genes most consistently implicated in ASD, like *PTEN, RELN, SYNGAP1, ANK2, SCN2A*, and *SHANK3*^51^. Noh et al. analysis of de novo CNVs spanning ASD genes also implicated cognitive processes, and showed a convergence in cellular component organization or biogenesis, cellular component assembly, and organelle organization biological processes^16^. Other studies implicated cell adhesion processes in ASD as important components of synapse formation and function^52,53^. Dysregulation of polyubiquitination was also in line with previous studies reporting an excess of variants in genes involved in ubiquitination processes, which regulate neurogenesis, neuronal migration, and synapse formation, and are thus essential for brain development^54–57^.

This biological heterogeneity parallels the extensive phenotypic heterogeneity that characterizes ASD. For this reason, we sought to identify the biological processes underlying the more homogeneous phenotypic subgroups defined by the clusters. The Random Forest algorithm was used to assess the importance of each enriched biological process in discriminating the two ASD phenotype subgroups. Feature importance analysis showed that all the biological processes contributed positively to the classification of ASD severity. However, the feature importance ranking was different from the significance ranking of enriched biological processes. Despite their relevance for ASD, the top three statistically significant biological processes identified by functional enrichment analysis were least important for the classification of subjects into the phenotypic milder and more dysfunctional subgroups. These findings support the concept that the integration of datasets with multidisciplinary information, including genomic and clinical data, is necessary to discover the biological mechanisms that lead to specific clusters of symptoms.

The Naive Bayes classifier was able to make useful predictions of ASD phenotypic subgroups from disrupted biological processes, but only for a subset of individuals for whom annotations had higher information content for the biological processes defined by their CNVs. Currently, GO contains more than 40,000 biological concepts, which are rapidly evolving with the increasing knowledge of biological phenomena, and with our ability to structure this knowledge. Therefore, it is expected that the performance of the proposed classifier will improve with the progress in GO annotations.

Given the high clinical heterogeneity of ASD, clustering of individuals according to a multidimensional phenotype will result in subgroups with more homogeneous clinical patterns, and for whom the causes of this disease are more likely to have the same underlying biological mechanism. The clustering of individuals according to multidimensional clinical symptoms *per se* is likely to have implications for prognosis and outcomes, as concurrent symptoms may have a synergistic effect on disease progression, and may thus also help guide clinical practice and intervention. However, thus far this perspective has been insufficiently explored, and not enough datasets are yet available with detailed clinical information that can be merged for large-scale analysis. The alterations in diagnostic criteria over time, and the changes in versions of instruments like the ADI-R and the ADOS, create important challenges for data merging across population samples, which are needed so that sufficient statistical power is achieved for definite conclusions. This study is clear in this limitation, as the number of subjects with important missing data in multiple clinical features was high in the AGP dataset, reducing analytical power. The next research step will necessarily have to involve overcoming limited clinical information and merging challenges between available datasets, like AGRE and the Simons Foundation Autism Research Initiative (SFARI), so that models established for biological predictions can be useful in clinical settings. On the other hand, while genomic information gets easier and cheaper to collect, improvements are also necessary regarding GO annotations, as a large number of subjects with phenotypic subgroup data did not have sufficient GO information content to be useful for classifier predictions.

## Conclusion

Overall, the present approach is proof of concept that genotype–phenotype correlations can be established in ASD, and that biological processes can predict multidimensional clinical phenotypes. Importantly, it highlights the usefulness of machine learning approaches that take advantage of multidimensional measures for the construction of more homogeneous clinical profiles. It further stresses the need to overcome the limitations of analyzing individual gene variants in favor of considering biological processes disrupted by a heterogeneous set of gene variants. The results stress two major requisites for translation of genomic information into useful clinical applications: that study datasets include detailed and complete clinical information, and that databases containing biological process information are rigorously and extensively curated. Overall, the methodology can be generalized to other datasets, including the clustering and classifier steps, provided that the necessary adaptations to data characteristics are considered. Identification of biological processes for specific clinical subgroups will be important to discover physiological targets for pharmacological therapy that can be efficient for subgroups of patients. This strategy can equally become very useful in clinical settings, for predicting outcomes and planning interventions for subgroups of patients whose specific patterns of clinical presentation are defined by the genes disrupted by specific genetic variants.

## Supplementary information

Supplementary file

## Data Availability

The code is available from the authors upon request.
